# Explainable AI in healthcare: a systematic review of XAI use cases in imaging, diagnostics, and rehabilitation

**DOI:** 10.3389/frai.2026.1749527

**Published:** 2026-04-01

**Authors:** Apoorva Aravindkumar, Marimuthu Ramadoss, Saqhibuddeen Ahmed Fakhruddin Ahmed, Vidhya Sampath, Kishor Lakshminarayanan

**Affiliations:** 1 School of Healthcare Science and Engineering, Vellore Institute of Technology, Vellore, Tamil Nadu, India; 2 School of Electronics Engineering, Vellore Institute of Technology, Vellore, Tamil Nadu, India

**Keywords:** clinical diagnosis, explainable artificial intelligence (XAI), Grad-CAM, LIME, medical imaging, rehabilitation, SHAP

## Abstract

**Background:**

Explainable artificial intelligence (XAI) is used in healthcare to make machine-learning outputs more transparent and clinically usable. This is important because many machine learning models work like a “black box” which can hide bias, reduce trust in the model. XAI addresses this problem by showing which features or image regions influenced a result, either for one patient or across a dataset.

**Objectives:**

Our objective is to provide a clear, systematic review of how XAI is being used in healthcare. We summarize the main XAI methods, the data and models they are paired with, and how these explanations support clinical understanding across imaging, diagnosis, and rehabilitation.

**Methods:**

We performed a systematic review with narrative synthesis (2020–2025) of 36 empirical studies across three verticals–Imaging (*n* = 10), Diagnosis (*n* = 16), and Rehabilitation (*n* = 10) that are identified via PubMed/MEDLINE, IEEE Xplore, and Google Scholar, following PRISMA 2020 guidelines. We included research studies that employed XAI in the three mentioned verticals. We excluded review articles and viewpoint studies. Screening numbers were - records identified 1,481; duplicates removed 647; other removals 187; screened 647; excluded 532; reports sought 115; not retrieved 31; assessed 84; full-text excluded 48; included 36. From each study we extracted ML models, XAI methods, study design, methodologies, and dataset/source. Meta-analysis was not undertaken due to heterogeneity.

**Results:**

Across 36 studies, SHAP was used in 21 studies, Grad-CAM in ~12/36, and LIME in ~11/36. A clear method-modality fit emerged with Imaging predominantly using saliency/heat-map methods, especially Grad-CAM, for spatial evidence. Diagnosis and Rehabilitation were dominated by feature-attribution tools like SHAP and LIME for global and case-level explanations. Many papers combined ≥ 2 explainers to cross-check interpretations namely SHAP+LIME, and Grad-CAM + LIME.

**Conclusion:**

Recent healthcare XAI demonstrates consistent method-modality fit and frequently combine two or more methods, helping translate opaque predictions into clinician-oriented reasoning. To enable trustworthy deployment, future work should pair these practices with standardized XAI reporting, faithfulness/stability assessments, and external, cross-site validation.

## 1 Introduction

### 1.1 Motivation

Artificial Intelligence (AI) and Machine Learning (ML) systems are increasingly applied to healthcare tasks medical imaging, clinical diagnosis, and risk prediction (Jiang et al., 2017). However, clinical deployment is uniquely sensitive because errors can directly affect patient outcomes, and because models need to be capable of generalization across hospitals, devices, populations, and clinical workflows (Shukla et al., 2025). High performance on internal test sets does not really translate to safe real-world use, particularly when training data can contain biases or site-specific patterns. This translational gap motivates approaches that support transparent model assessment and responsible deployment, including explainable AI (Loh et al., 2022).

AI refers to the ability of machines to perform tasks associated with human intelligence (Sheikh et al., 2023). Early framing such as the Turing Test was historically influential (Turing, 1950), and later work debated the limits of imitation as a marker of intelligence (Saygin et al., 2000; Epstein et al., 2007; Hoffmann, 2022; Mitchell, 2024) In modern AI, ML and Deep Learning (DL) have emerged as two crucial paradigms for real-world applications. ML refers to a class of algorithms that learn relationships from training data for automation, decision-making, and prediction (Janiesch et al., 2021), DL is a subset of ML that uses artificial neural networks to learn underlying representations (Balyen and Peto, 2019). The shift from rule-based systems to data-driven learning has enabled AI to expand into practical, high-impact domains, particularly healthcare (Jiang et al., 2017; Shukla et al., 2025; Sengupta and Lakshminarayanan, 2024; Lakshminarayanan et al., 2024).

The early reference of AI being used in healthcare was in the 1960s with the development of rule-based expert systems such as MYCIN which offered diagnostic and treatment suggestions for infectious diseases, and CASNET, which was used for glaucoma diagnosis (Coles, 1977). Through the 1990s, AI methods shifted towards early applications in imaging and electronic health records (Sharma, 2020). With the advent of DL in the 2010s, the arena expanded to radiology, dermatology and ophthalmology (Hirani et al., 2024). Despite early promise, adoption of many early systems was limited in practice due to challenges such as limited validation, difficulty integrating into real clinical workflows, and governance/transparency concerns. These issues remain closely tied to explainability and accountability requirements in recent times.

### 1.2 Problem statement

Despite the impressive performance of AI systems, the black-box nature of these AI systems poses significant risks. Several studies have demonstrated that models can arrive at correct predictions through wrong assumptions and reasons that resemble more a shortcut than a thoughtful pattern. Studies have shown AI systems relying on artifacts notably hospital tags, rulers in images or dataset-specific biases to arrive at the conclusion. Zech et al. (2018) demonstrated that Convolutional Neural Networks (CNNs) trained to detect pneumonia from chest X-rays were able to identify hospital-specific metal tokens that were a part of the X-ray image and used them as proxies for deciding the disease prevalence rather than relying on the radiographic pathology. Though the overall accuracy was high, it was a result of poor external generalization. A similar case of AI taking shortcuts in decision making is when Winkler et al. (2019) explored the diagnostic performance of AI for melanoma detection and found that the model focused on contextual artifacts such as rulers used for lesion measurement rather than skin lesion features, clearly showing how context cues can mislead AI models. DeGrave et al. (2021) showed that DL models developed to detect COVID-19 from chest X-rays used non-clinical signals such as text labels, laterality tokens, metadata rather than lung pathology. Another similar example from Coudray et al. (2018) also showed that the AI models could exploit image-level artifacts or staining patterns which are not related to tumor biology while trying to classify lung cancer subtypes. These examples underscore why failures of generalization and false correlations are particularly consequential in healthcare, where incorrect reasoning can remain hidden behind high accuracy and may lead to unsafe deployment. Such limitations highlight the need for approaches that help interrogate model behavior and decision rationale.

Explainable artificial intelligence (XAI) is a feature or capability of AI systems that explains the rationale behind the decisions made by the AI models. DL models such as Deep Neural Networks (DNNs) used for crucial decision-making applications achieved high predictive accuracy in complex areas such as image processing, diagnosis and natural language processing (Kabir et al., 2025). However, the lack of interpretability made these AI models essentially a “black-box.” XAI aims to reduce model opacity and improve transparency and accountability. However, explainability alone does not guarantee clinical trustworthiness. Explanations can also be misleading if they are unstable, unfaithful to the model’s true reasoning, or not evaluated in real clinical contexts.

### 1.3 Research gap

Several reviews have summarized XAI in healthcare by grouping studies according to explanation method families or data modalities (Loh et al., 2022; Srinivasu et al., 2022; Sheu and Pardeshi, 2022; Sadeghi et al., 2023; Band et al., 2023; Kalasampath et al., 2025; Chaddad et al., 2023). These perspectives provide a useful foundation for understanding the landscape of techniques. However, existing reviews rarely organize evidence around concrete clinical use cases or examine how explanations are situated within imaging, diagnostic, and rehabilitation workflows, including how they are evaluated and reported. In particular, there is limited synthesis of how XAI methods align with modality and task, how explanation quality and stability are assessed, and how often human-centered or translational outcomes are reported. This review addresses that gap by providing a use-case-based synthesis that emphasizes where explanations appear in clinical pathways, how they interact with end users, and what is known about their evaluation and reporting practices across imaging, diagnosis, and rehabilitation.

### 1.4 Scope of this review

To address this gap, we conducted a systematic review of empirical XAI applications in three critical healthcare verticals: medical imaging, clinical diagnosis, and rehabilitation. We included peer-reviewed studies published between 2020 and 2025 that applied any XAI technique to real-world human health data and reported model performance and explanation outputs. The review focuses on (i) which XAI methods are used with which data types and model families, (ii) how explanations are reported and, when available, evaluated, and (iii) what methodological, ethical, and reporting gaps may limit translation to clinical practice. Meta-analysis was not undertaken due to substantial heterogeneity in tasks, models, datasets, and endpoints.

### 1.5 Paper organization

This paper is structured as follows. Section 2 outlines literature survey, background concepts and terminology relevant to explainable AI in healthcare. Section 3 details the methodology, including information sources, search strategy, eligibility criteria, study selection, data extraction, and risk-of-bias assessment. Section 4 presents the results and thematic synthesis across imaging, diagnosis, and rehabilitation. Section 5 discusses implications for translation, limitations, identified research gaps, and future research directions. Section 6 outlines future research directions in more detail. Section 7 concludes the paper.

## 2 Background

### 2.1 Literature survey

XAI in healthcare sits at the intersection of long-standing work on AI in medicine and more recent advances in model interpretability. Early expert systems such as MYCIN and CASNET demonstrated that symbolic rule-based systems could provide transparent diagnostic suggestions in infectious diseases and glaucoma, but they were limited by brittle knowledge bases and narrow coverage (Coles, 1977; Sharma, 2020). The subsequent shift to data-driven machine learning and deep learning enabled substantial gains in accuracy for tasks such as radiology, dermatology, and ophthalmology (Jiang et al., 2017; Sengupta and Lakshminarayanan, 2024; Lakshminarayanan et al., 2024; Hirani et al., 2024). However, these models typically operate as black boxes and offer limited insight into their decision processes, raising concerns about generalization across hospitals, devices, and populations and motivating renewed interest in explainability as a condition for trustworthy deployment in safety-critical settings (Jiang et al., 2017; Shukla et al., 2025; Loh et al., 2022).

Several methodological surveys have catalogued XAI techniques and taxonomies relevant to healthcare. These reviews often distinguish between model-agnostic versus model-specific methods, *post-hoc* versus intrinsic interpretability, and global versus local explanations (Loh et al., 2022; Kabir et al., 2025; Srinivasu et al., 2022; Sheu and Pardeshi, 2022; Sadeghi et al., 2023; Band et al., 2023; Kalasampath et al., 2025; Chaddad et al., 2023). Model-agnostic approaches such as LIME and SHAP approximate a complex model’s behavior using surrogate models or game-theoretic feature attributions, enabling flexible application across different classifiers and data types (Sadeghi et al., 2023; Ribeiro et al., 2016; Lundberg and Lee, 2017). Model-specific methods including Grad-CAM, Integrated Gradients, Layer-wise Relevance Propagation (LRP), and related saliency techniques exploit internal network structure or gradients to produce relevance maps, particularly for convolutional neural networks in imaging tasks (Selvaraju et al., 2017; Sundararajan et al., 2017; Wachter et al., 2018; Bach et al., 2015). Intrinsic models-such as sparse linear models, decision trees, and rule-based systems-remain attractive for their inherent interpretability but may sacrifice performance for transparency in complex clinical settings, especially when compared with high-capacity neural networks and ensembles (Puthanveettil Madathil et al., 2024; Rudin, 2019). Collectively, this body of work establishes the technical foundations of XAI but is often agnostic to concrete clinical workflows and translational constraints.

Domain-focused reviews and case studies have begun to explore XAI applications in specific areas such as radiology, oncology, neurology, and electronic health records (Loh et al., 2022; Srinivasu et al., 2022; Sheu and Pardeshi, 2022; Sadeghi et al., 2023; Band et al., 2023; Kalasampath et al., 2025; Chaddad et al., 2023). In medical imaging, recent empirical work commonly combines deep convolutional architectures with saliency or heatmap-based explanations to highlight disease-relevant regions in modalities such as radiography, CT, and MRI. For instance, Grad-CAM has been used to visualize the regions driving classifications in kidney CT, chest radiographs for pneumonia and COVID-19, and brain MRI or SPECT for neurological disorders, often with the goal of enhancing clinician trust by aligning highlighted regions with known pathology patterns (Zech et al., 2018; Winkler et al., 2019; DeGrave et al., 2021; Coudray et al., 2018; Selvaraju et al., 2017; Abir et al., 2024; Ferdous et al., 2024; Hasan et al., 2024; Haque et al., 2025; Turkan and Tek, 2021; Noun et al., 2025). Glioma-focused pipelines further combine Grad-CAM or related heatmaps with model-agnostic methods such as SHAP and LIME to connect subtype predictions and prognostic estimates to radiogenomic markers and anatomically meaningful MRI regions (Tupe-Waghmare et al., 2021; Palkar et al., 2024; Yoon and Lin, 2025; Pilaoon et al., 2025). However, many imaging studies report explanation outputs only qualitatively, relying on visual plausibility or anecdotal clinician feedback without systematic evaluation of explanation faithfulness, stability, or robustness (Abir et al., 2024; Ferdous et al., 2024; Hasan et al., 2024; Turkan and Tek, 2021; Noun et al., 2025; Tupe-Waghmare et al., 2021; Palkar et al., 2024; Yoon and Lin, 2025; Pilaoon et al., 2025). This limits the ability to judge whether heatmaps genuinely reflect model reasoning or merely provide reassuring post-hoc narratives.

In clinical diagnosis and risk prediction, XAI has been applied extensively to tabular and mixed-modal datasets. Studies using tree-based ensembles, gradient boosting methods, logistic regression, and neural networks for tasks such as breast and cervical cancer detection, multi-marker biosensing, lung cancer risk stratification, autism and epilepsy diagnosis, and stroke-risk prediction frequently employ SHAP to generate both global feature importance profiles and local case-level attributions (Lundberg and Lee, 2017; Tong et al., 2025; Hariprasad et al., 2024; Tanim et al., 2024; Yukta et al., 2024; Choi et al., 2025; Radhakrishnan et al., 2024; AlMohimeed et al., 2023; Shakil et al., 2024; Wani et al., 2024; Sebastian et al., 2025; Suryavanshi and Kukreja, 2025; Sujana and Augustine, 2023; Paolucci et al., 2023; Ahmad et al., 2024; Shobana et al., 2025). LIME is often used alongside SHAP to provide complementary local explanations, while permutation feature importance (PFI), Random Forest feature importance (RFI), and Anchors offer more classical or rule-based measures of variable contribution (Sadeghi et al., 2023; Tong et al., 2025; Hariprasad et al., 2024; Tanim et al., 2024; AlMohimeed et al., 2023; Shakil et al., 2024; Sujana and Augustine, 2023; Ahmad et al., 2024; Shobana et al., 2025). A subset of diagnostic work also leverages model-specific explainers such as Grad-CAM, Integrated Gradients, and saliency maps when deep learning models are used for image-based oncology or neuroimaging tasks (Selvaraju et al., 2017; Radhakrishnan et al., 2024; Narasimha Raju et al., 2025; Suryavanshi and Kukreja, 2025). Despite this activity, most diagnostic studies still focus evaluation on conventional performance metrics such as accuracy, F1-score, and AUC, while explanation evaluation-when reported-is limited to descriptive summaries, small-scale clinician feedback, or isolated case illustrations rather than standardized quantitative assessment of faithfulness or stability (Hariprasad et al., 2024; Tanim et al., 2024; Yukta et al., 2024; Choi et al., 2025; Radhakrishnan et al., 2024; AlMohimeed et al., 2023; Shakil et al., 2024; Narasimha Raju et al., 2025; Liu et al., 2024; Wani et al., 2024; Sebastian et al., 2025; Suryavanshi and Kukreja, 2025; Sujana and Augustine, 2023; Paolucci et al., 2023; Ahmad et al., 2024; Shobana et al., 2025).

Rehabilitation and longitudinal care form a relatively newer but growing area for XAI. In these studies, models are typically built on structured data from demographics, clinical scores, wearable sensors, motion capture, gait and fatigue measurements, or ground reaction forces, often using regression or classification to predict functional outcomes, therapy response, or risk of adverse events (Gandolfi et al., 2023; Lee and Choy, 2023; Lee et al., 2024; Abdelaziz et al., 2025; Gupta et al., 2025; Hussain and Jany, 2024; Slijepcevic et al., 2023; Slijepcevic et al., 2024). Random-forest and boosting-based prognosis models for upper-limb recovery and safe balance are frequently paired with SHAP, LIME, PFI, and RFI to assess feature relevance and cross-method consensus, clarifying which baseline measures and imaging-derived biomarkers drive predicted outcomes (Gandolfi et al., 2023; Lee et al., 2024). Sensor- and video-based rehabilitation pipelines use gradient-based saliency maps or 1D Grad-CAM over time-series networks to localize compensatory movements and gait deviations, while Anchors and TreeSHAP provide rule-like or tree-specific attributions over EMG, IMU, and other biomechanical features that therapists can audit at the task level (Lee and Choy, 2023; Abdelaziz et al., 2025; Hussain and Jany, 2024; Slijepcevic et al., 2023; Slijepcevic et al., 2024). Beyond stroke, EMG- and PPG-based fatigue prediction and exercise-personalization studies employ SHAP and related attribution methods to link neuromuscular and autonomic markers or multi-marker improvements to predicted fatigue states and sarcopenia risk, supporting individualized adjustment of interventions in aging populations (Gupta et al., 2025; Guo et al., 2024; He et al., 2024). Nevertheless, formal evaluation of explanation stability over time, robustness to sensor noise and protocol variation, and practical impact on clinical decision-making remains limited, with few rehabilitation studies incorporating rigorous user-centered or decision-impact evaluations involving clinicians and patients (Gandolfi et al., 2023; Lee and Choy, 2023; Lee et al., 2024; Abdelaziz et al., 2025; Gupta et al., 2025; Hussain and Jany, 2024; Slijepcevic et al., 2023; Slijepcevic et al., 2024; Guo et al., 2024; He et al., 2024).

Across these verticals, existing reviews and empirical applications collectively highlight several recurring gaps. First, most syntheses are organized around XAI method families or data modalities rather than concrete clinical use cases or end-to-end workflows, making it difficult to compare how explanations are actually used in imaging, diagnostic, and rehabilitation settings (Loh et al., 2022; Srinivasu et al., 2022; Sheu and Pardeshi, 2022; Sadeghi et al., 2023; Band et al., 2023; Kalasampath et al., 2025; Chaddad et al., 2023). Second, evaluation of explanation quality is often under-specified: while many papers present heatmaps or feature importance plots, relatively few report systematic measures of faithfulness, stability, robustness, or human interpretability, and even fewer evaluate how explanations affect clinician performance, calibration, or workflow efficiency (Abir et al., 2024; Ferdous et al., 2024; Hasan et al., 2024; Turkan and Tek, 2021; Noun et al., 2025; Tupe-Waghmare et al., 2021; Palkar et al., 2024; Yoon and Lin, 2025; Pilaoon et al., 2025; Hariprasad et al., 2024; Tanim et al., 2024; Yukta et al., 2024; Choi et al., 2025; Radhakrishnan et al., 2024; AlMohimeed et al., 2023; Shakil et al., 2024; Narasimha Raju et al., 2025; Liu et al., 2024; Wani et al., 2024; Sebastian et al., 2025; Suryavanshi and Kukreja, 2025; Sujana and Augustine, 2023; Paolucci et al., 2023; Ahmad et al., 2024; Shobana et al., 2025; Gandolfi et al., 2023; Lee and Choy, 2023; Lee et al., 2024; Guo et al., 2024; He et al., 2024). Third, bias and fairness considerations are inconsistently addressed; subgroup analyses, differential explanation patterns across demographics or sites, and structured mitigation strategies are rarely reported in a systematic way (Shukla et al., 2025; Loh et al., 2022; Kabir et al., 2025; Sadeghi et al., 2023; Zhou et al., 2024; Lipton, 2018; Ciatto et al., 2020). Finally, rehabilitation-focused XAI remains comparatively underexplored despite its potential for personalized, adaptive interventions in chronic and age-related conditions (Gandolfi et al., 2023; Lee and Choy, 2023; Lee et al., 2024; Abdelaziz et al., 2025; Gupta et al., 2025; Hussain and Jany, 2024; Slijepcevic et al., 2023; Slijepcevic et al., 2024; Guo et al., 2024; He et al., 2024).

The present review builds on this literature by providing a use-case-based synthesis of XAI applications in three key healthcare verticals: medical imaging, clinical diagnosis, and rehabilitation. Rather than focusing solely on algorithms or data types, we organize evidence around where and how explanations are embedded in clinical pathways, what types of models and XAI methods are used for each vertical, and how explanation outputs are reported and evaluated (Abir et al., 2024; Ferdous et al., 2024; Hasan et al., 2024; Haque et al., 2025; Turkan and Tek, 2021; Noun et al., 2025; Tupe-Waghmare et al., 2021; Palkar et al., 2024; Yoon and Lin, 2025; Pilaoon et al., 2025; Hariprasad et al., 2024; Tanim et al., 2024; Yukta et al., 2024; Choi et al., 2025; Radhakrishnan et al., 2024; AlMohimeed et al., 2023; Shakil et al., 2024; Narasimha Raju et al., 2025; Liu et al., 2024; Wani et al., 2024; Sebastian et al., 2025; Suryavanshi and Kukreja, 2025; Sujana and Augustine, 2023; Paolucci et al., 2023; Ahmad et al., 2024; Shobana et al., 2025; Gandolfi et al., 2023; Lee and Choy, 2023; Lee et al., 2024; Abdelaziz et al., 2025; Gupta et al., 2025; Hussain and Jany, 2024; Slijepcevic et al., 2023; Slijepcevic et al., 2024; Guo et al., 2024; He et al., 2024). By systematically comparing method-modality fit, validation designs, explanation evaluation practices, and ethical and reporting considerations across these verticals, this review aims to clarify the current state of XAI in healthcare and identify concrete methodological and translational gaps that future work needs to address (Loh et al., 2022; Kabir et al., 2025; Srinivasu et al., 2022; Sheu and Pardeshi, 2022; Sadeghi et al., 2023; Band et al., 2023; Kalasampath et al., 2025; Chaddad et al., 2023; Page et al., 2021; Moons et al., 2025).

### 2.2 XAI explained

#### 2.2.1 Explainability

Interpretability refers to the ability to follow the decision-making process of a model inherently without having a need to explain. For example, simple models such as decision trees are easily interpretable on their own. However, explainability refers to the capability of any model to be able to explain why the decision was made in human-understandable form. This is usually needed when complexity and size of the model grows, and it becomes difficult to understand how the model is interpreting the decision. For example, neural networks, random forests can interpret the decision making at a smaller scale but as the model gets bigger it is no longer interpretable (Zhou et al., 2024; Lipton, 2018). While “explainability” and “interpretability” are often found to be used interchangeably in the literature, several studies point this out to be an existing ambiguity in defining the objectives and scope of XAI (Kabir et al., 2025; Zhou et al., 2024; Ciatto et al., 2020).

#### 2.2.2 Classification

Model agnostic are general purpose methods that can be applied to a wide range of ML models regardless of their structure or training mechanism. This is because they treat the model as a black-box and analyze only the inputs and outputs. Examples of model-agnostic methods include Local Interpretable Model-Agnostic Explanations (LIME) and Shapley Additive Explanations (SHAP) (Sadeghi et al., 2023). LIME, first proposed in 2016, is a model that explains the individual predictions for an instance by approximating the local decision boundary of any opaque model. The specific instance of the input data is perturbed using local surrogate models to explain the model’s behavior. The LIME model acts as a foundational model that led to various metrics being developed for human-centered interpretability (Ribeiro et al., 2016). In contrast, SHAP, developed in 2017, applies cooperative game theory with ML models for interpretability. Each feature is assigned Shapley values which acts as an importance value based on the fair share of contributions made by the feature. SHAP is theoretically grounded and consistent, and used for structured or tabular data explanations mostly (Lundberg and Lee, 2017). Because LIME and SHAP only require inputs and outputs, the same explanation workflow can be applied to both a random forest and a neural network for Emergency Department deterioration risk without accessing model internals.

Model specific explanations are designed to work only with specific algorithms as they exploit the internal structure of the training model (Sadeghi et al., 2023). As a result, they lack generalization and cannot be transferred across models. For DL models in imaging, Grad-CAM presented in 2016, helps visualize where a CNN “looks” when it is making a decision. It computes the gradients, calculates weights, combines activations and overlays heatmaps on the original image. This is done by tracing gradients to the last convolutional layer, revealing class-relevant spatial regions. This is a model-specific method used for CNNs and hence is widely adopted in medical imaging and diagnosis (Selvaraju et al., 2017).

Intrinsic or ante-hoc tools are interpretable models that use inherently transparent and simple models such as decision trees, linear regression and rule-based models. The computational structure of these models make them intrinsically interpretable; however the structure can impact the accuracy of these models too (Puthanveettil Madathil et al., 2024). For example, an early sepsis risk can be directly read using a sparse logistic regression on the patient vitals and lab reports making it interpretable by design (Rudin, 2019). Post-hoc XAI models explain the black-box models after the decision is made. These are retroactively deployed after the prediction is made. Post-hoc models are more adaptable as they can be applied to any pre-trained model (Madsen et al., 2022). A classic example would be an XGBoost model that predicts heart-failure readmission. An XAI model (SHAP) can attribute the factors behind the prediction thereby giving the risk factors for heart-failure (Lundberg and Lee, 2017).

Global explanations describe how the model works as a whole focusing on overall feature importance, structural behavior and decision boundaries of a model. An example would be a model describing age, blood pressure and cholesterol as top predicting factors for coronary heart diseases (Tong et al., 2025). Local explanations on the other hand point out why the model made a specific prediction for a single case. For example, in a healthcare setting, local methods similar to LIME may point out why a patient was flagged as high-risk for cancer. However, they lack generalization and one patient’s outcome cannot be applied to another (Sadeghi et al., 2023).

Integrated Gradients (IG) is an interpretability technique or method that attributes the output of a DNN to its input features. It improves upon raw gradient methods by calculating the integral of gradients from a baseline to the actual input. IG was built to satisfy two main axioms: sensitivity–where if changing a feature can change the prediction, that feature must get non-zero attribution and implementation invariance - where two models that compute the same function receive the same attributions, regardless of how they are implemented. Because of these guarantees and its simplicity, IG is widely used in practice, including medical image classification and other sequence or spatio-temporal tasks (Sundararajan et al., 2017).

Similarly, Counterfactual Explanations (CE) provide “what-if” scenarios by identifying the minimal changes in input features that can alter a model’s prediction. This method, instead of explaining the working of the black-box model, gives a minimal set of changes to the input feature and checks if that would alter the output. This model provides the user meaningful insights without having the need to open an entire black-box (Wachter et al., 2018).

Another neural method, Layer-wise Relevance Propagation (LRP), shows how much each input feature has contributed to the output by back propagating the output relevance score through the network layers and redistributing the relevance to each input feature. This is a conservative method that works for deep networks (Bach et al., 2015).

Lastly, anchors which were developed as an extension of LIME are high-precision, rule-based explanations that ensure that when certain conditions (referred to as anchors) hold true for a model, the output remains the same. Anchors are more precise as they come with an empirical estimation of reliability. Anchors are more human-interpretable as the rules are simple “if-then” statements that can be easily understood (Ribeiro et al., 2018). These methods collectively form the backbone of modern XAI, enabling transparency and interpretability across a range of healthcare applications, from diagnostic imaging to rehabilitation.

## 3 Methodology

### 3.1 Study design and protocol

We conducted an extensive systematic search of the existing literature on XAI in healthcare focusing on three main verticals - Medical Imaging, Diagnosis, and Rehabilitation. These three verticals were defined *a priori* based on the vast expanse of applications and based on the intended scope of our study to form a crucial end-to-end pathway from perception (image acquisition and interpretation), through decision-making (patient-level diagnostic reasoning), to intervention and follow-up (therapy planning and monitoring). The studies were assigned to a primary vertical based on the objective and the primary outcome mentioned in the study. When the objective was ambiguous, then the classification was done based on the clinical context. We also focused on the aspect of translational readiness in order to prioritize areas that can change clinical decisions or clinician’s behavior. Within this frame, the review aims to describe how XAI is used alongside ML models, summarize reported validation practices and evaluation metrics, and identify recurring ethical and reporting gaps that may limit adoption. We did not attempt a meta-analysis due to the significant heterogeneity in study design, including variations in tasks, models, datasets, outcomes, and clinical contexts. Specifically, differences in data types, model architectures, explanation methods, evaluation metrics, and clinical endpoints made it impractical to pool results meaningfully.

### 3.2 Information sources and search strategy

We searched PubMed/MEDLINE, IEEE Xplore, and Google Scholar for articles published between 2020 and 2025. The date of final literature search was on 18th of October 2025. Search strings combined domain terms with XAI terminology. Boolean operators were used systematically: OR broadened the search to capture synonyms and related terms, AND narrowed results to studies clearly situated in healthcare and explicitly employing XAI. We tried truncation and wildcards; however, these introduced substantial noise, often fetching studies outside healthcare, so we did not rely on them in the final queries. Database filters were applied to restrict the time window to 2020–2025 and to exclude review and viewpoint articles. For Google Scholar, where ranking is relevance-based and result volumes are high, screening was limited to the first two pages (first 20 results) per query to reduce noise while prioritizing the most relevant records. For transparency, we organized the search strategy by vertical (Imaging, Diagnosis, Rehabilitation). Table 1 provides an overview of the keyword blocks used for each vertical, while the complete database-specific search strings for PubMed/MEDLINE, IEEE Xplore, and Google Scholar are reported in Supplementary Table S1 to improve reproducibility. (Table 1).

**Table 1:** Systematic search strategy for identifying literature on applications of explainable AI in medical imaging, diagnosis, and rehabilitation.

Verticals	Keywords	Queries
Imaging	XAI in medical imaging, Explainable AI for image analysis, AI in MRI, AI in CT scan, Grad-CAM in radiology, Explainability in radiology, AI in medical imaging interpretation	(“explainable artificial intelligence” OR “interpretable AI”) AND (“imaging”) (“Grad-CAM” OR “class activation map*” OR SHAP OR “explainable AI” OR “explainable artificial intelligence” OR “XAI”) AND (MRI OR “magnetic resonance” OR “brain CT” OR stroke OR tumor) NOT (“Detection” or “Diagnosis”)
Diagnosis	XAI in diagnosis, XAI for medical diagnosis, AI in cardiology, XAI in oncology, XAI in neurology, XAI risk assessment healthcare	(“explainable artificial intelligence” OR “interpretable AI”) AND (“diagnosis” OR “medical diagnosis”)
Rehabilitation	XAI in rehabilitation, AI in rehabilitation devices, XAI for wearable sensors, rehabilitation AI, rehabilitation robotics XAI	(“explainable artificial intelligence” OR “interpretable AI”) AND (“rehabilitation”) (“Explainable AI” OR “AI in rehabilitation”) AND (“wearable sensors” OR “rehabilitation robotics” OR “assistive devices” OR “rehabilitation technologies”)

**Figure 1:**
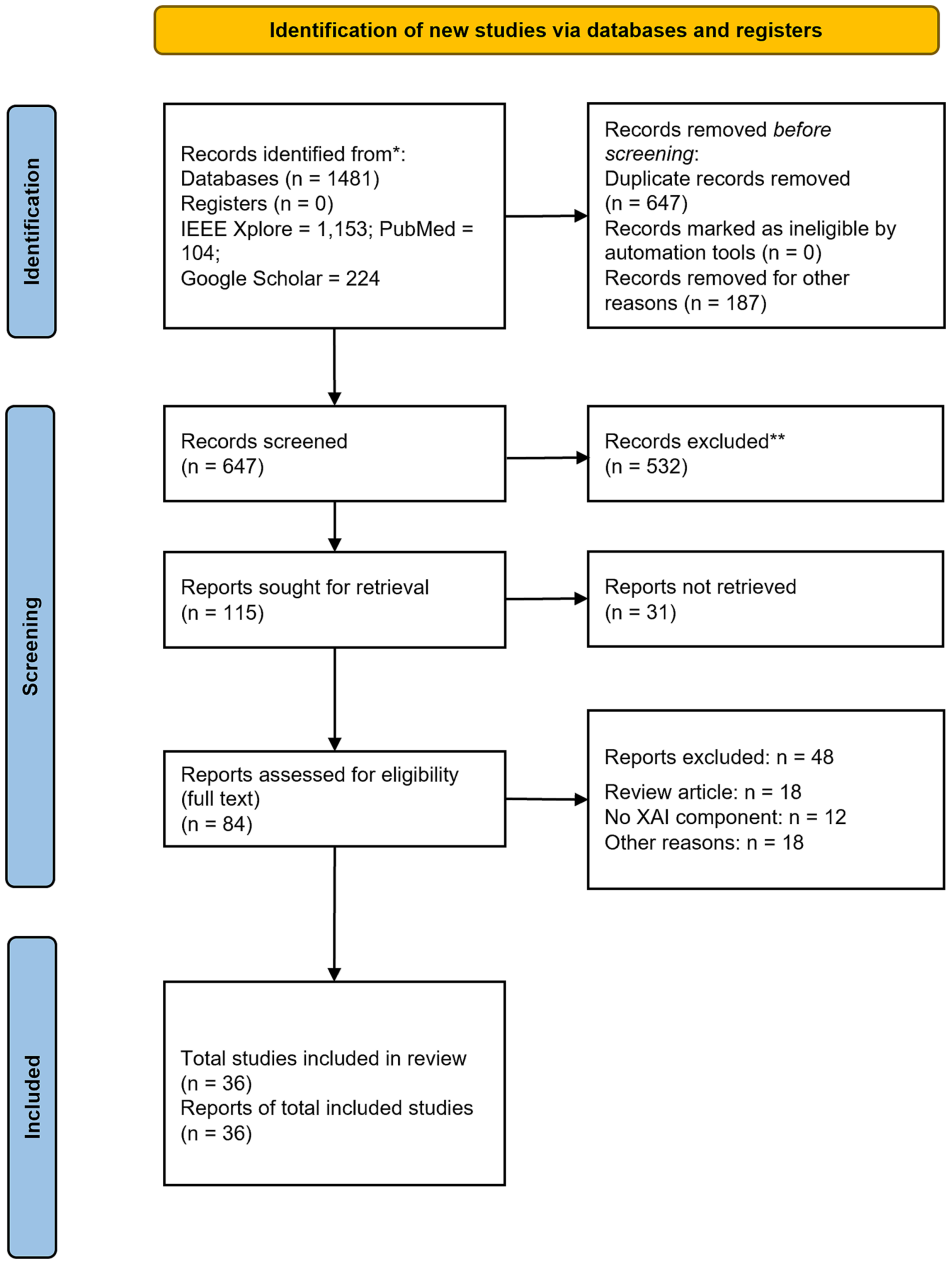
PRISMA 2020 flow diagram (databases and registers only). Records identified from databases (*n* = 1,481); duplicates removed (*n* = 647); records removed for other reasons (*n* = 187); records screened (*n* = 647); records excluded (*n* = 532); reports sought (*n* = 115); reports not retrieved (*n* = 31); reports assessed (*n* = 84); full-text excluded with reasons (*n* = 48); studies included (*n* = 36). Adapted from PRISMA 2020 (Page et al., 2021).

Eligibility criteria were defined using the PICOS framework. We included studies involving human healthcare data (Population), reporting machine-learning models that incorporated any XAI technique (Intervention). A comparator was not required for inclusion (Comparator), as the primary aim of this review was synthesize XAI applications by use-case rather than to compare them with benchmark models or explainers. Studies were required to report model performance and description of explanations (Outcomes) and use empirical designs with real patient datasets (Study design). We excluded review articles, viewpoints, studies that did not satisfy the inclusion requirements, and all records published before 2020. The review was restricted to studies published in the English language. This resulted in obtaining a contemporary sample of studies focused on concrete applications rather than conceptual overviews. We followed the PRISMA 2020 guidelines and presented the study selection in Figure 1 (Page et al., 2021).

### 3.3 Data extraction

For each included study, two reviewers extracted bibliographic information, the clinical domain and task, dataset details (source and sample population), ML model(s) employed, XAI method(s) used, how explanations were evaluated when reported, and validation design. Any disagreements between the reviewers were resolved through discussion, and adjudicated by a third reviewer if a mutual decision was not reached. We also recorded performance metrics such as accuracy, precision, recall, F1-score, Area Under the Curve (AUC)/Receiving Operating Characteristic (ROC), and regression measures such as Root Mean Squared Error (RMSE) or Mean Absolute Percentage Error (MAPE). Finally, we noted ethics and reporting elements including statements on fairness or bias, informed consent or Institutional Review Board (IRB) approval, and any explicit use of reporting standards or checklists. All extracted fields were cross verified against the original papers by a third reviewer to minimize transcription or interpretation error.

### 3.4 Outcomes and synthesis

Tasks, inputs, and outcome definitions varied widely due to which we synthesized findings narratively, grouping studies by the three verticals and, within each, by sub-domain. Sub-domains were defined *a priori* based on the primary clinical context and data type reported in each study: imaging studies were grouped by modality/organ system (e.g., radiography vs. neuroimaging), diagnosis studies by condition area (e.g., oncology vs. neurological disorders), and rehabilitation studies by population and application focus (e.g., stroke vs. gait/fatigue vs. aging/intervention personalization). Studies spanning multiple sub-domains were assigned to the sub-domain most aligned with the primary dataset and outcome, and noted as multi-domain when applicable. We paid particular attention to method-modality fit where imaging studies tended to favor saliency or heatmap-based approaches such as Grad-CAM. Comparatively, diagnosis and rehabilitation frequently employed tabular or mixed data and often used feature-attribution methods such as SHAP and, at times, LIME. We also summarized the evaluation metrics most frequently reported. Ethical and reporting practices were noted as described in each paper. Given the heterogeneity in designs and endpoints, we did not pool effect sizes and performed a narrative synthesis.

### 3.5 Data management and tools

Records were captured and managed using reference-management software (Zotero and CiteDrive). Citation chaining was performed using an automated literature-mapping tool (Research Rabbit). Screening, data extraction, and tabulation were performed in shared spreadsheets, and manuscript drafting and collaborative annotation were conducted in shared documents (Google Sheets and Google Docs).

### 3.6 Risk of bias and applicability

The PROBAST + AI tool was used to assess the quality, risk of bias, and applicability of healthcare prediction models of the eligible studies (Moons et al., 2025). PROBAST-AI evaluates four risk-of-bias domains (Participants and Data Sources, Predictors, Outcome, Analysis) and three applicability domains (Participants, Predictors, Outcome) using structured signaling questions. Two reviewers independently completed the PROBAST-AI assessment for each study, and disagreements were resolved through discussion, with adjudication by a third reviewer when needed. Signaling questions were recorded as yes/probably yes, no/probably no, or no information, and domain judgments were assigned as low, high, or unclear risk/concern following PROBAST-AI guidance. Overall risk of bias was judged high if any risk-of-bias domain was rated high, unclear if one or more domains were unclear and none were high, and low only when all domains were low. Applicability was judged relative to alignment with the review’s intended clinical setting.

The assessment showed that many included studies exhibited high or unclear concern in one or more methodological domains. This reflects the evolving nature of XAI in research in healthcare. The analysis domain accounted for most of these concerns (high concern: 30/36; unclear: 4/36; low: 2/36), due to modest sample sizes, limited or absence of external validation (0/36 studies reported external validation), and incomplete reporting of model development procedures which are all common challenges in early-stages of machine-learning. In the Participants and Data Sources domain, several studies were based on single-center or retrospective datasets, which naturally restrict generalizability and are typical of preliminary exploratory investigations. Predictors and outcomes were generally described clearly although in some cases reporting gaps existed (Predictors: low 29/36; unclear 7/36; Outcomes: low 12/36; unclear 23/36; high 1/36). Applicability assessments similarly reflected the limitations such as the impact of narrower population or data characteristics affecting broader clinical transferability (overall applicability: low 9/36; high 10/36; unclear 17/36). Overall, findings show that, XAI is advancing, but methodological and reporting improvements are still needed.

## 4 Results

### 4.1 Descriptive overview of the included studies

A total of 36 studies were included across three verticals (Imaging: *n* = 10; Diagnosis: *n* = 16; Rehabilitation: *n* = 10). Across these included studies, the most frequently used XAI methods were SHAP (~21 studies), Grad-CAM (~12 studies), and LIME (~11 studies). Other notable methods reported include Anchors, permutation/Random-Forest importance, Integrated Gradients, DeepLIFT, saliency, LRP, occlusion, 3D-UCM, and QLattice. Many studies used more than one explainer methods (e.g., SHAP + LIME; Grad-CAM + LIME; Integrated Gradients + Grad-CAM + DeepLIFT), indicating a frequent approach in using more than one explainer rather than relying on a single method.

Most studies, particularly in imaging and diagnosis, used publicly available datasets for model development and explanation. Reported train/test split was 80:20, followed by 70:30 and 75:25, with some studies using three-way splits such as 70:15:15, 70:20:10, and 75:10:15. Several studies did not clearly report split ratios. Cross-validation practices were inconsistent: 5-fold and 10-fold CV were more frequently reported, with occasional use of 2-, 3-, or 7-fold CV, and some studies not reporting cross-validation. Performance evaluation most often included accuracy, F1-score, precision, and recall. However, confusion matrices and error-rate measures (e.g., FPR/FNR) were less consistently reported. Rehabilitation studies predicting continuous outcomes commonly reported regression metrics such as RMSE and MAPE.

### 4.2 Explainable artificial intelligence in medical imaging

#### 4.2.1 Radiography

A lightweight CNN was deployed along with three post-hoc XAI methods–LIME, SHAP, and Grad-CAM in a study (Abir et al., 2024) to classify Computed Tomography (CT) images of kidneys into normal, cyst, tumor, and stone. A huge CT kidney dataset of 12,446 images (normal–3709, cyst–5077, tumor–1377, and stone–2283) was downloaded from Kaggle. LIME was used as a local and model-agnostic tool to approximate the behavior of the model within the influential image regions for each individual case. SHAP, also model-agnostic, provided both local and global attributions, showing the features that the model relied on across the dataset and for specific images. In order to visualize the influential regions within each image, heat-maps were generated through a model-specific XAI method, Grad-CAM. The authors reported that these explanations could improve transparency and clinician acceptance. However, the paper did not report quantitative evaluation of explanation faithfulness/stability or other reliability checks, and evaluation focused on classifier performance.

In another instance, XAI was used to visualize the areas in chest X-ray images that were used for classifying the images into normal, COVID-19, and pneumonia (Ferdous et al., 2024). The dataset comprised 21,000 images in total merged from 4 public data sets and used a collection of transfer-learning models, namely MobileNetV2, ResNet-50/152, Vision Transformer (ViT), and a custom CNN. Grad-CAM was used as a post-hoc and local method to show the regions of the image that the ensemble model focused on. The XAI method here, assigns weights to the feature map by calculating the gradients of the output to the last convolutional layer. This resulted in a heat map which was overlaid on the original image to visually show the areas that were of high importance. The study also used a custom tool called GradCAMCallback to save these Grad-CAM images after each epoch. Though the study states that the visualizations generated by the XAI method enhanced the understanding of the model’s behavior, there are no metrics to evaluate the Grad-CAM method.

Similarly, Grad-CAM heatmaps were used to classify radiology images where a new model named CTXNET, a compact transformer-CNN hybrid derived from Compact Convolutional Transformer (CCT), was used (Hasan et al., 2024). Chest X-rays (21,149 images) and CT (194,919 images) were augmented to ~49 k images via DCGAN (Deep Convolutional Generative Adversarial Network) and contained normal, COVID-19, lung opacity, and viral pneumonia cases. Grad-CAM heatmaps visualized class-relevant regions for clinician interpretation.

A CNN-attention model for lung cancer classification was enhanced by combining SHAP and Grad-CAM with a Gradio web interface (Haque et al., 2025). SHAP, as a post-hoc model-agnostic method, provided both local and global explanations by assigning Shapley values to input features or pixels, using a kernel explainer for case-level attributions and summary and force plots to reveal dataset-level importance patterns. Grad-CAM, as a post-hoc model-specific technique for CNNs, computed class-targeted gradients over the last convolutional layer to produce class-discriminative heatmaps that highlight image regions most influential to the decision, emphasizing positively contributing areas via ReLU and upsampling the activation map for visualization. Gradio enables real-time, user-friendly interaction with the model, allowing clinicians to upload images, view predictions, and inspect explanations, and the study presented this interface as facilitating clinician-facing review and supporting transparency and usability in clinical assessment.

#### 4.2.2 Neuroimaging

A Visual Geometry Group (VGG)-based 3D CNN pipeline for Alzheimer’s Disease (AD) Magnetic Resonance Imaging (MRI) classification incorporated four post-hoc XAI techniques - Occlusion, 3D Ultrametric Contour Map (3D-UCM), 3D Grad-CAM, and SHAP-to interrogate model behavior on class decisions (Turkan and Tek, 2021). Occlusion systematically masked patches in the volume to assess sensitivity of predictions to localized signal, while 3D-UCM provided boundary-aware segmentations that helped compare saliency with neuroanatomical structure. 3D Grad-CAM generated class-specific heat-maps from the final convolutional layers to highlight discriminative regions, and SHAP offered additive feature attributions to quantify contribution patterns across inputs. Together, these methods were used to compare an enhanced VoxCNN16 against an attention-augmented variant (VoxATT), with the explanations illustrating where each model focused when separating Alzheimer’s Disease (AD) from NC and EMCI, and helping to rationalize the incremental gains attributed to added filters and attention.

For Parkinson’s Disease (PD) classification from DaTSCAN SPECT, a 3D CNN was paired with Grad-CAM to visualize class-relevant uptake patterns across the volume (Noun et al., 2025). Grad-CAM saliency was used as a local, post-hoc, model-specific explainer, producing heat-maps that traced the network’s decision evidence beyond canonical striatal regions. The resulting maps suggested attention over cortical and sub-cortical territories in addition to the striatum, which the authors interpreted as indicating decision evidence beyond canonical striatal regions and as potentially consistent with broader PD-related signatures. In this framing, Grad-CAM served both as a plausibility check (are highlighted areas clinically sensible?) and as a hypothesis-generating tool for exploring non-striatal involvement.

Across glioma MRI from 2021 to 2025, studies increasingly fused multimodal modeling with complementary XAI to align predictions with radiogenomic markers and clinician intuition (Tupe-Waghmare et al., 2021; Palkar et al., 2024; Yoon and Lin, 2025; Pilaoon et al., 2025). Early semi-supervised, multi-task approaches used visual explanations (e.g., Grad-CAM) to relate subtype predictions to anatomically meaningful MRI regions (Tupe-Waghmare et al., 2021). Subsequent “transparent pipeline” work combined classical learners with model-agnostic XAI-SHAP for global and local feature attributions, LIME for local surrogate explanations, and related tools–to highlight the influence of key genomic or imaging features at both cohort and case levels (Palkar et al., 2024). Later semi-supervised models again leveraged Grad-CAM to connect molecular subtype outputs with recognizable radiogenomic patterns in multimodal MRI (Yoon and Lin, 2025). Most recently, hybrid ensemble CNNs paired Grad-CAM and LIME to provide complementary saliency and perturbation-based views of tumor-influenced regions, emphasizing clinician-facing interpretability alongside high discriminative performance (Pilaoon et al., 2025). Collectively, these works position Grad-CAM for visual localization, SHAP for principled attribution summaries, and LIME for case-wise reasoning. Yet, across these studies, reporting of explanation faithfulness, stability, and external validation was limited.

### 4.3 Explainable artificial intelligence in clinical diagnosis

This section reviews XAI for clinical diagnosis across oncology and neurological conditions, highlighting how studies applied feature-attribution methods and case-level explanations to interpret model outputs for risk categorization and diagnostic decision support.

#### 4.3.1 Oncology

In breast cancer diagnosis, recent work has paired strong predictors with post-hoc explainers so clinicians can see why a label was produced. A comparative study highlighted tree-based models and used SHAP to verify that medically sensible variables (e.g., menopausal status, tumor size) drove decisions, while LIME supplied per-case rationales to support bedside interpretation (Hariprasad et al., 2024). A deep-learning counterpart reached similar accuracy and likewise combined SHAP (global and local attributions) with LIME (instance-level surrogates), foregrounding interpretability alongside performance to aid clinical acceptance (Tanim et al., 2024).

For renal cancer, CNNs were coupled with complementary XAI views to expose image-evidence behind predictions: Grad-CAM produced heat-maps localizing class-relevant regions, and LIME offered case-wise, model-agnostic explanations to sanity-check individual decisions (Yukta et al., 2024). In prostate cancer, a biosensing + ML pipeline used logistic regression over multi-marker features and applied Kernel SHAP to quantify how each biomarker (and PI-RADS) shifted the decision boundary - turning a black-box score into an additive, evidence-weighted narrative clinicians could audit (Choi et al., 2025).

Beyond these, ovarian cancer studies tested multiple CNN backbones and layered several attribution methods - Integrated Gradients, Saliency, Grad-CAM, DeepLIFT - to triangulate which structures drove multiclass judgments and to compare consistency across explainers (Radhakrishnan et al., 2024). For early cervical cancer detection, tabular pipelines emphasized robust feature handling and then used SHAP to deliver both global importance profiles and subject-level explanations, complementing sampling strategies (e.g., SMOTE/ADASYN) and feature selection (e.g., χ^2^, RFE, LASSO) with interpretable outputs that clinicians can scrutinize (AlMohimeed et al., 2023; Shakil et al., 2024). Across these threads, SHAP anchors global + local attributions for tabular and DL features, LIME supplies human-readable local surrogates, and Grad-CAM (with IG/Saliency/DeepLIFT) visualizes image evidence–though most papers still report metrics for the classifier, not for explanation faithfulness or stability, underscoring the need for standardized XAI evaluations and external validation.

Two recent studies in the field of skin cancer use XAI to improve diagnosis and risk communication. Raju et al. introduce XAI-SkinCADx, a six-stage, dermoscopy-driven framework that combines intrinsic and post-hoc explainability, where Grad-CAM++ is applied immediately after the combined CNN block using the DenseNet201 conv5_block32_concat layer to produce local saliency maps via higher-order gradients, and LIME interrogates the final DM-25 + BiLSTM + SVM ensemble by perturbing super-pixels configured with 1,000 samples, positive-only explanations, and five super-pixels; which the authors described as supporting clinician confidence and interpretability of the diagnostic output (Narasimha Raju et al., 2025). Liu et al. employ a local, *post-hoc*, example-based approach on 2D facial images, selectively decoding CPH-derived facial endophenotypes to generate synthetic image sequences spanning low to high risk, which links input parameters to risk scores and allows visual and manual inspection of salient attributes such as lower BMI, reduced skin smoothness, and increased facial erythema. The study presented this as a way to support interpretability without modifying the predictor (Liu et al., 2024).

Across two complementary studies on lung cancer prediction with a hybrid ConvXGB model, Wani et al. and Sebastian et al. use XAI to enhance the model behavior for clinical use. Wani et al. center DeepXplainer on SHAP, a *post-hoc*, model-agnostic method that provides local and global interpretability by quantifying each feature’s contribution via Shapley values, using SHAP. DeepExplainer with force plots to clarify individual cases and SHAP summary plots with clustering to surface dataset-level importance patterns and trends that guide medical judgment (Wani et al., 2024). Likewise, Sebastian et al. employ SHAP to highlight key predictors such as ESR and to articulate diagnostic rationale at both local and global levels, and further incorporate Diverse Counterfactual Explanations (DiCE) to analyze misclassifications by generating multiple diverse, plausible counterfactuals that reveal sources of confusion and identify minimal feature changes needed to flip incorrect decisions, thereby strengthening interpretability, error analysis, and overall clinical trustworthiness of the ConvXGB approach (Sebastian et al., 2025).

#### 4.3.2 Neurological disorders

A hybrid Convolutional Neural Network-Vision Transformer (CNN-ViT) framework for Alzheimer’s Disease incorporated SHAP, Grad-CAM, and LIME to deliver complementary explanations across global, regional, and case-level views (Suryavanshi and Kukreja, 2025). Data were drawn from ADNI with four classes (NC, MAD, SAD; overall size not explicitly reported). Reported performance (accuracy 92.5%, F1 91.2%, AUC 94%) was contextualized by explanation use: SHAP summarized feature contributions (global/local), Grad-CAM localized class-relevant neuroanatomy, and LIME provided instance-wise surrogates to rationalize subtype discrimination and low misclassification.

For Autism Spectrum Disorder, phenotypic predictors (PIQ, VIQ, FIQ, ADOS, ADI) were paired with SHAP, LIME, and Anchors to expose both dataset-level and individual decision logic, clarifying which attributes most influenced classification (Sujana and Augustine, 2023). Video-based pre-screening leveraged an XGBoost classifier with SHAP values and beeswarm plots to rank interaction features, embedded in a rigorous validation design (5-fold nested, stratified child-based CV; inner hyperparameter tuning; outer unbiased estimation; repeated ten times) that linked explanation patterns to clinically recognizable body-related signs (e.g., attunement) and yielded robust AUCs across independent raters (Paolucci et al., 2023).

Interpretable EEG seizure detection emphasized justification alongside accuracy via a stacking ensemble (Bagging RF, Decision Tree, XGBoost) and SHAP for both cohort-level summaries and per-case attributions that clinicians can audit; datasets spanned Bonn, UCI, and CHB-MIT, with privacy-preserving governance described via a blockchain layer (Ahmad et al., 2024).

In stroke-risk modeling on a class-imbalanced tabular dataset (mitigated with SMOTE), comparative baselines (logistic regression, SVM, RF, KNN, DT) were augmented with SHAP, LIME, and ELI5 to reveal global and case-level drivers - consistently implicating age, BMI, and glucose-and to relate directionality (e.g., increasing age ↔ higher risk) to clinical priors; logistic regression emerged strongest and improved with hyperparameter tuning (Shobana et al., 2025). Across these neuro/behavioral and tabular-clinical contexts, SHAP reliably anchors global + local attribution for structured features, LIME (and Anchors) supports case-level rationales, and Grad-CAM provides spatial grounding for imaging; yet, explanation faithfulness/stability metrics, bias assessment, and prospective, real-world validation remain underreported.

### 4.4 Explainable artificial intelligence in rehabilitation

Rehabilitation represents a distinct XAI paradigm compared with diagnosis because the clinical goal is typically longitudinal decision support rather than a single time-point classification. Models are used to monitor recovery, forecast trajectories, and adapt interventions across repeated sessions, often using heterogeneous functional measurements (e.g., assessment scores, kinematics, EMG/IMU, video, GRF) collected under changing conditions. So, explainability in rehabilitation should focus on practical guidance (what the clinician should do next), consistency over time (explanations that stay similar or change in a meaningful way across sessions), and reliability when conditions change - for example due to fatigue, learning effects, sensor placement differences, or changes in the patient’s impairment. Explanations also need to be interpretable to both clinicians and patients to support shared goal-setting and therapy adjustment, making workflow integration and human-centered evaluation especially central in rehabilitation-focused XAI.

Stroke rehabilitation currently dominates the XAI-rehab literature. Random-forest-based prognosis modeling has been paired with SHAP, LIME, permutation feature importance (PFI), and Random Forest-specific interpretability (RFI) to assess feature relevance and cross-method consensus for upper-limb recovery, with XAI surfaces clarifying which baseline measures drive predicted outcomes (Gandolfi et al., 2023). In sensor- and video-based upper-limb training, saliency maps over feed-forward networks highlight frame-level compensations; gradient-based scoring and thresholding isolate only the video segments requiring therapist review, reducing labeling burden while keeping decisions auditable (Lee and Choy, 2023). Prognostic modeling for safe balance similarly combines model comparison with permutation importance and SHAP to show that Fugl-Meyer motor scores and ipsilesional corticospinal tract integrity are consistently dominant predictors, linking explanations to actionable clinical constructs (Lee et al., 2024). Wearable, small-N, exercise-specific pipelines use Anchors to generate rule-like, human-checkable conditions over EMG/Kinect features, supporting per-task decisions during Wolf Motor Function Test exercises (Abdelaziz et al., 2025).

Beyond stroke, EMG- and PPG-based fatigue prediction integrates SHAP on optimized classifiers to attribute neuromuscular and autonomic markers (e.g., EMG spectral and amplitude features; PPG variability) to predicted fatigue states, enabling clinicians to validate whether the model’s emphasis matches physiological expectations for internal versus external rotation tasks (Gupta et al., 2025). In gait analysis, TreeSHAP/LIME/Anchors explain boosting and forest models that separate stroke from healthy controls, repeatedly surfacing right-side spectral EMG features (e.g., biceps femoris, lateral gastrocnemius) as strong discriminators during ambulation (Hussain and Jany, 2024). Large-scale GRF modeling combines linear SVMs and CNNs with Layer-wise Relevance Propagation (LRP) to localize person-specific and pathology-robust signatures around GRF peaks - particularly anteroposterior force - supporting individualized therapy tuning (Slijepcevic et al., 2023). For cerebral palsy gait, time-series Grad-CAM (adapted to 1D) and model-intrinsic importance reveal that traditional models attend predominantly to clinically emphasized regions, while deep nets leverage both canonical and additional kinematic cues; kinematic signals outperform GRF for subtype classification in this setting (Slijepcevic et al., 2024).

XAI has also been applied to exercise-intervention personalization in aging populations. For instance, strength training combined with traditional modalities (e.g., Tai Chi, Yi Jin Jing) is linked to reversal of sarcopenia risk through SHAP-based attribution of multi-marker improvements, and, when paired with Markov Chain attribution, yields individualized, temporally coherent explanations of intervention effects to guide program adjustments (Guo et al., 2024; He et al., 2024). Across these threads, attribution methods (SHAP, LIME, Anchors, PFI/RFI) consistently elevate clinically recognized predictors and expose case-level rationale; localization methods (saliency, Grad-CAM, LRP) verify that models attend to plausible biomechanical or neurophysiological signals in videos, kinematics, EMG, and GRF. Overall, XAI in rehabilitation presently adds value to prognosis and intervention support by aligning predictions with measurable constructs and therapist heuristics, but wider adoption will depend on standardized faithfulness/stability reporting, bias assessment, and prospective, real-world evaluations integrated into routine therapy planning.

## 5 Discussion

In the Results section, we summarized key study characteristics such as data source, model family, XAI approach, and the reported evaluation metrics. We provided consolidated tables of ML models and explainability methods (Tables 2–4).

**Table 2:** Consolidated list of ML algorithms and XAI methods from the included studies in medical imaging.

Subsection	Author(s)	ML algorithm used	XAI method used
Radiography	Abir et al. (2024)	Lightweight CNN	SHAP, LIME, Grad-CAM
	Ferdous et al. (2024)	Ensemble of MobileNetV2, ResNet-50/152, ViT, custom CNN	Grad-CAM
	Hasan et al. (2024)	CTXNET (compact transformer-CNN hybrid from CCT)	Grad-CAM
	Haque et al. (2025)	CNN	SHAP, Grad-CAM
Neuroimaging	Turkan and Tek (2021)	3D VGG variants (VoxCNN16, VoxATT)	Occlusion, 3D-UCM, 3D Grad-CAM, SHAP
	Noun et al. (2025)	3D CNN	Grad-CAM
	Tupe-Waghmare et al. (2021)	Semi-supervised multi-task deep learning	HR-CAMs
	Palkar et al. (2024)	XGBoost, SVM (transparent pipeline)	SHAP, LIME, QLattice
	Yoon and Lin (2025)	Semi-supervised multi-task deep learning	Grad-CAM
	Pilaoon et al. (2025)	Ensemble CNNs	Grad-CAM, LIME

**Table 3:** Consolidated list of ML algorithms and XAI methods from the included studies in clinical diagnosis.

Subsection	Author(s)	ML algorithm used	XAI method used
Oncology	Hariprasad et al. (2024)	Random Forest	SHAP, LIME
	Tanim et al. (2024)	DL (unspecified architecture)	SHAP, LIME
	Yukta et al. (2024)	CNN	Grad-CAM, LIME
	Choi et al. (2025)	Logistic Regression (with multi-marker inputs)	Kernel SHAP
	Radhakrishnan et al. (2024)	Seven DL models (incl. InceptionV3, MobileNetV2, VGG19, ResNet18, ResNeXt, Xception, EfficientNet)	Integrated Gradients, Saliency Map, Grad-CAM, DeepLift
	AlMohimeed et al. (2023)	Stacked ensemble (meta-learner over multiple ML models)	SHAP
	Shakil et al. (2024)	Decision Tree (with Chi-square; other preprocessing noted)	SHAP
	Narasimha Raju et al. (2025)	Hybrid Feature Extraction, CNN, BiLSTM, Multiclass SVM, Deep Ensemble Learning	Grad-CAM++, LIME
	Liu et al. (2024)	ANN based Autoencoder, CPH	Example-based custom XAI approach, Selective Decoding
	Wani et al. (2024)	ConvXGB (CNN + XGBoost)	SHAP
	Sebastian et al. (2025)	ANN, DNN	SHAP, Counterfactual Explanations
Neurological disorders	Suryavanshi and Kukreja (2025)	Hybrid CNN-ViT	SHAP, Grad-CAM, LIME
	Sujana and Augustine (2023)	DL model (unspecified)	SHAP, LIME, Anchors
	Paolucci et al. (2023)	XGBoost	SHAP (values and beeswarm plots)
	Ahmad et al. (2024)	Stacking ensemble classifier (Bagging RF, DT, XGB)	SHAP (global and local)
	Shobana et al. (2025)	Logistic regression best (compared to SVM, RF, KNN, DT)	SHAP, LIME, ELI5

**Table 4:** Consolidated list of ML algorithms and XAI methods from the included studies in rehabilitation.

Subsection	Author(s)	ML algorithm used	XAI method used
Rehabilitation	Gandolfi et al. (2023)	Random Forest regression	SHAP, LIME, PFI, RFI
	Lee and Choy (2023)	Feed-forward neural network	Saliency map (plus gradient-based and threshold-based methods)
	Lee et al. (2024)	Multiple compared; Random Forest best	Permutation importance, SHAP
	Abdelaziz et al. (2025)	Random Forest, SVM	Anchors
	Gupta et al. (2025)	Compared RF, SVM, LR, DT, KNN (SHAP on optimized KNN)	SHAP
	Hussain and Jany (2024)	Gradient Boosting, Histogram Gradient Boosting, Random Forest	SHAP (TreeSHAP), LIME, Anchors
	Slijepcevic et al. (2024)	Linear SVM (best), also RF, CNN, MLP, DT	LRP
	Slijepcevic et al. (2023)	CNNs, SNNs, RFs, DTs (RF best for certain angles)	Grad-CAM (1D), Gini feature importance
	Guo et al. (2024)	Stacking model and LDA, DT, GBC, LightGBM, ETC, XGBoost, LR, RF, KNN	SHAP
	He et al. (2024)	LDA, DT, GBC, LightGBM, ETC, XGBoost, LR, RF	SHAP

### 5.1 Explainability patterns by modality

Across the three verticals, explainability choices showed a consistent alignment with data modality. Imaging studies predominantly used saliency or heatmap based explanations, with Grad-CAM frequently applied, which may reflect the practical fit of spatial heatmaps for image classification tasks. In imaging, SHAP was reported on instances when classical ML models such as SVM, and XGBoost were used. In contrast, clinical diagnosis studies more often applied feature-attribution methods such as SHAP, frequently alongside LIME, and typically with decision trees, random forests, and other ensemble models. A smaller subset of diagnosis that used DL or CNNs employed IGs, Grad-CAM, and saliency maps. This may be influenced by the convenience of feature-attribution methods for comparing contributions across heterogenous model families. Rehabilitation studies relied primarily on structured measurements namely demographics, questionnaires, assessment scores, sensor data, and SHAP was commonly used with linear and ensemble models to generate both local and global attributions. Several studies (Noun et al., 2025; Narasimha Raju et al., 2025; Liu et al., 2024; Ahmad et al., 2024; Guo et al., 2024) mentioned some form of explanation evaluation or clinician feedback. However, none of these studies included formal clinician evaluation or systematic validation of explanation faithfulness. While algorithmic explanations, such as SHAP feature importance and Grad-CAM heatmaps, were commonly reported, most studies did not validate these explanations in terms of their clinical relevance, accuracy, or correctness. This highlights a significant gap in the current literature, where most evaluations focus primarily on model performance, rather than on validating the faithfulness or clinical relevance of the explanations provided.

We also observed limited adoption of transformer-based and semi-supervised approaches in a small number of studies, most notably within imaging, In addition, post-hoc explainers such as SHAP were sometimes applied even when the study used inherently interpretable models, indicating that the explainability was frequently implemented as an additional post-hoc layer and that using a model-agnostic model is uncommon.

### 5.2 Limitations

The findings of this review should be interpreted in the light of the following limitations. The scope of the review is limited to three databases which gives a possibility of missing out on studies available in other sources. Some databases and journals could not be accessed due to Paywall restrictions. The English-only review may have missed non-English studies that could have contributed to the field of research. Since XAI methods are rapidly evolving, certain terminologies or some very recent approaches may not be fully captured. Our review discusses selected examples from each vertical in detail hence may not have covered every available paper in each domain. These constraints mean that the review is best interpreted as a structured synthesis of prevailing trends across the three verticals, rather than a consolidation of all XAI studies in healthcare.

## 6 Future research directions

Future research should prioritize strengthening test reliability and generalizability of XAI-enabled healthcare models. Instead of assuming that high accuracy and visually plausible explanations will transfer to new inputs or clinical settings, upcoming studies should systematically test sensitivity to small input perturbations, missing values, and measurement noise, and evaluate whether both performance and explanation behavior remain stable under dataset shifts across sites, scanners, acquisition protocols, demographics, and devices. This includes prioritizing external and cross-site validation or other strong distribution-shift evaluations, and reporting not only changes in conventional performance metrics but also whether explanation patterns change in meaningful ways (for example, drift in highlighted regions or features, inconsistent feature attributions, or altered rationales under similar clinical scenarios).

Methodologically, future work should document validation designs with greater precision, including train/test split strategies, cross-validation schemes (k, stratification, subject-wise versus sample-wise splitting, and use of nested CV where appropriate), and explicit evaluation of explainability beyond narrative claims. Researchers should specify how transparency was operationalized and whether faithfulness, stability, and robustness checks were performed, ideally guided by structured reporting frameworks such as CONSORT-AI and SPIRIT-AI when relevant. This level of rigor is particularly important in rehabilitation, where data are longitudinal and patients’ status evolves over time; here, explanations should be evaluated for consistency across sessions and robustness to fatigue, learning effects, sensor placement variability, and changing impairment levels to ensure that they support therapy adjustment rather than reflect transient artifacts.

Addressing bias and usability should also be central in future XAI studies. Investigators should routinely assess and mitigate bias by reporting subgroup performance (for example, by age, sex, site, device, or protocol), examining whether explanation patterns differ systematically across subgroups, and documenting mitigation steps such as balanced sampling, reweighting, calibration, domain adaptation, or post-deployment monitoring when disparities are detected. Finally, evaluations should move beyond simply stating that “an explainer was included” to demonstrating that explanations are interpretable and actionable for clinicians, supported by interface designs that enable efficient review, and validated through usability-oriented evidence such as clinician feedback, task-based experiments, or decision-impact studies that show explanations meaningfully aid decision-making rather than adding complexity.

## 7 Conclusion

This review aims to synthesize how XAI is being applied across the three verticals of healthcare such as medical imaging, clinical diagnosis, and rehabilitation. It also identifies recurring method choices and evaluation practices that influence translation to practice. Across the studies included, Grad-CAM is most frequently used in imaging, while SHAP and LIME dominate diagnosis and rehabilitation. However, it is also noted that many of the studies use a combination of methods notably SHAP + LIME, SHAP + LIME + Grad-CAM, and Grad-CAM + IG to cross-check insights. Because the evidence base was heterogeneous in tasks, datasets, models, and evaluation, this synthesis is best interpreted as a structured overview of current practice, and it remains limited by restricted database coverage, English-only inclusion, and inconsistent reporting of explanation faithfulness/stability and clinical workflow impact. In this context, trustworthy clinical practice is defined operationally as evidence of robust external validation, transparent reporting, reliability checks for explanations, and human-centered evaluation demonstrating safe workflow integration. Overall, prioritizing interpretability alongside validation, bias mitigation, privacy, and clinician-facing assessment outlines a realistic path for moving XAI from promising prototypes toward trustworthy clinical practice.

## Data Availability

The original contributions presented in the study are included in the article/Supplementary material, further inquiries can be directed to the corresponding author/s.

## Glossary

1D (Grad-CAM): One-Dimensional (time-series)
3D-UCM 3D: Ultrametric Contour Map
AD: Alzheimer’s Disease
ADASYN: Adaptive Synthetic Sampling
ADNI: Alzheimer’s Disease Neuroimaging Initiative
AI: Artificial Intelligence
Anchors: XAI method name; rule-based “anchors”
AUC/AUROC: Area Under the ROC Curve
CCT: Compact Convolutional Transformer
CE: Counterfactual Explanations
CHB-MIT: Children’s Hospital Boston-MIT EEG Database
CNN/CNNs: Convolutional Neural Network(s)
CT: Computed Tomography
CTXNET: (Model name) Compact Transformer-CNN Hybrid
DCGAN: Deep Convolutional Generative Adversarial Network
DL: Deep Learning
DNNs: Deep Neural Networks
DT: Decision Tree
ELI5: “Explain Like I’m 5” (explainability toolkit)
EMCI: Early Mild Cognitive Impairment
EMG: Electromyography
ETC: Extra Trees Classifier
F1-score: F1-score (harmonic mean of precision and recall)
FET: Field-Effect Transistor (dual-gate FET biosensor)
GBC: Gradient Boosting Classifier
Grad-CAM: Gradient-weighted Class Activation Mapping
GRF: Ground Reaction Force
HR-CAMs: High-Resolution Class Activation Maps
IG: Integrated Gradients
IRB: Institutional Review Board
KNN: k-Nearest Neighbors
LASSO: Least Absolute Shrinkage and Selection Operator
LDA: Linear Discriminant Analysis
LightGBM: Light Gradient Boosting Machine
LIME: Local Interpretable Model-Agnostic Explanations
LR: Logistic Regression
LRP: Layer-wise Relevance Propagation
MAPE: Mean Absolute Percentage Error
ML: Machine Learning
MLP: Multilayer Perceptron
NC: Normal Controls
PD: Parkinson’s Disease
PFI: Permutation Feature Importance
PI-RADS: Prostate Imaging Reporting and Data System
PPG: Photoplethysmography
QLattice: Symbolic regression-based model search framework
RF: Random Forest
RFE: Recursive Feature Elimination
RFI: Random Forest Feature Importance
RMSE: Root Mean Squared Error
ROC: Receiver Operating Characteristic
SHAP: Shapley Additive Explanations
SNNs: Spiking Neural Networks
SPECT: Single-Photon Emission Computed Tomography
SMOTE: Synthetic Minority Over-sampling Technique
SVM: Support Vector Machine
STFT: Short-Time Fourier Transform
TreeSHAP: Tree-specific SHAP implementation
TT: Turing Test
UCI: University of California, Irvine (ML Repository)
VGG: Visual Geometry Group (network family)
ViT: Vision Transformer
XAI: Explainable Artificial Intelligence
XGBoost: Extreme Gradient Boosting
