# Supplementary material for: Explainable AI in healthcare: a systematic review of XAI use cases in imaging, diagnostics, and rehabilitation
Source: Front Artif Intell. 2026 Apr 1;9:1749527. doi: 10.3389/frai.2026.1749527 (PMC13079713; doi:10.3389/frai.2026.1749527)
Supplement: Supplementary file 1 [file Table_1.DOCX]

# Search Results and Database Queries

In response to the reviewer’s request for reproducibility, we have reproduced the search queries used in the systematic review. Supplementary Table S1 presents the general search blocks used for the literature searches in PubMed/MEDLINE, IEEE Xplore, and Google Scholar. These search blocks were applied with specific filters (e.g., year range 2020-2025, English language) to ensure that the searches were consistent and reproducible.

## PubMed/MEDLINE

| **Vertical** | **Exact search string** | **Filters** |
| --- | --- | --- |
| Imaging | ("explainable artificial intelligence"[Title/Abstract] OR "explainable AI"[Title/Abstract] OR XAI[Title/Abstract] OR interpretability[Title/Abstract] OR explainability[Title/Abstract]) AND ("medical imaging"[Title/Abstract] OR radiology[Title/Abstract] OR MRI[Title/Abstract] OR "magnetic resonance"[Title/Abstract] OR CT[Title/Abstract] OR "computed tomography"[Title/Abstract] OR ultrasound[Title/Abstract] OR "X-ray"[Title/Abstract] OR mammography[Title/Abstract] OR OCT[Title/Abstract]) | Year: 2020–2025; Language: English |
| Diagnosis | ("explainable artificial intelligence"[Title/Abstract] OR "explainable AI"[Title/Abstract] OR XAI[Title/Abstract] OR interpretability[Title/Abstract] OR explainability[Title/Abstract]) AND (diagnosis[Title/Abstract] OR "medical diagnosis"[Title/Abstract] OR "clinical decision support"[Title/Abstract] OR CDSS[Title/Abstract] OR prognosis[Title/Abstract] OR "risk prediction"[Title/Abstract] OR "clinical prediction"[Title/Abstract] OR triage[Title/Abstract]) | Year: 2020–2025; Language: English |
| Rehabilitation | ("explainable artificial intelligence"[Title/Abstract] OR "explainable AI"[Title/Abstract] OR XAI[Title/Abstract] OR interpretability[Title/Abstract] OR explainability[Title/Abstract]) AND (rehabilitation[Title/Abstract] OR physiotherapy[Title/Abstract] OR "physical therapy"[Title/Abstract] OR "occupational therapy"[Title/Abstract] OR "rehabilitation robotics"[Title/Abstract] OR exoskeleton[Title/Abstract] OR "assistive device"[Title/Abstract] OR "wearable sensors"[Title/Abstract] OR IMU[Title/Abstract] OR gait[Title/Abstract] OR "motor recovery"[Title/Abstract]) | Year: 2020–2025; Language: English |

## IEEE Xplore

| **Vertical** | **Exact search string** | **Filters** |
| --- | --- | --- |
| Imaging | ("explainable AI" OR "explainable artificial intelligence" OR XAI OR interpretability OR explainability) AND ("medical imaging" OR radiology OR MRI OR "magnetic resonance" OR CT OR "computed tomography" OR ultrasound OR "X-ray" OR mammography OR OCT) | Years: 2020–2025; English; Journals & Conferences |
| Diagnosis | ("explainable AI" OR "explainable artificial intelligence" OR XAI OR interpretability OR explainability) AND (diagnosis OR "clinical decision support" OR CDSS OR prognosis OR "risk prediction" OR triage) | Years: 2020–2025; English; Journals & Conferences |
| Rehabilitation | ("explainable AI" OR "explainable artificial intelligence" OR XAI OR interpretability OR explainability) AND (rehabilitation OR "rehabilitation robotics" OR exoskeleton OR "assistive device" OR "wearable sensors" OR IMU OR gait OR "motor recovery") | Years: 2020–2025; English; Journals & Conferences |

## Google Scholar

| **Vertical** | **Exact search string** | **Filters** |
| --- | --- | --- |
| Imaging | ("explainable AI" OR "explainable artificial intelligence" OR XAI OR interpretability OR explainability) ("medical imaging" OR radiology OR MRI OR "magnetic resonance" OR CT OR "computed tomography" OR ultrasound OR "X-ray" OR mammography OR OCT) | Custom range: 2020–2025; patents & citations excluded; first 2 pages |
| Diagnosis | ("explainable AI" OR "explainable artificial intelligence" OR XAI OR interpretability OR explainability) (diagnosis OR "clinical decision support" OR CDSS OR prognosis OR "risk prediction" OR triage) | Custom range: 2020–2025; patents & citations excluded; first 2 pages |
| Rehabilitation | ("explainable AI" OR "explainable artificial intelligence" OR XAI OR interpretability OR explainability) (rehabilitation OR "rehabilitation robotics" OR exoskeleton OR "assistive device" OR "wearable sensors" OR IMU OR gait OR "motor recovery") | Custom range: 2020–2025; patents & citations excluded; first 2 pages |

^[[1]](#endnote-1)^ Google Scholar results are relevance-ranked and may vary over time; therefore, exact query strings, date limits, and screening depth (first two pages) are reported to enhance transparency.

1. [↑](#endnote-ref-1)
